# Diseases of the Ear, Their Diagnosis and Treatment

**Published:** 1864-05

**Authors:** 


DISEASES OF THE EAR, THEIR DIAGNOSIS AND TREATMENT. A Text-Book of Aural Surgery, in the form of Academical Lectures. By Dr. ANTON VON TROLTSCH, Aural Surgeon and Lecturer in the University, in Wurzburg, Bavaria. Translated from the German and Edited by D. B. ST. JOHN ROOSA, M.D., Assistant-Surgeon to the New York Eye Infirmary. Illustrated with Wood Engravings. From the Second and last German Edition. New York: WM. WOOD & Co., 61 Walker street. 1864.

This is a well executed volume of 254 pages, on one of the most neglected, and yet one of the most important, departments of practical medicine and surgery. It is a plain practical treatise on diseases of the Ear, in the style of lectures; sufficiently full in details without being prolix, and its practical recommendations characterized by clearness and good sense. The editor, Dr. ROOSA, has not only performed the work of translation well, but he has added many valuable paragraphs to the original text. The book is worthy of being purchased and read carefully, by every student and practitioner of medicine.

